# Wearable low-level laser therapy (laser acupuncture) versus manual acupuncture for chronic insomnia: protocol for a randomized, assessor-blinded, superiority trial

**DOI:** 10.3389/fpsyt.2026.1814245

**Published:** 2026-06-01

**Authors:** Yuchuan Shen, Hao Wen, Tao Kong, Junru Lin, Ai Chen, Yuyan Huang, Yanzhao Lin, Wei Bin

**Affiliations:** 1 The Second Affiliated Clinical Medical College, Guangzhou University of Chinese Medicine, Guangzhou, China; 2 Chinese Medicine Guangdong Laboratory (Hengqin Laboratory), Zhuhai, China

**Keywords:** acupuncture, chronic insomnia, heart rate variability, laser acupuncture, low-level laser therapy, photobiomodulation, randomized controlled trial, study protocol

## Abstract

**Background:**

Chronic insomnia is a prevalent and disabling condition associated with impaired daytime functioning, psychiatric comorbidity, and autonomic dysregulation. Although cognitive behavioral therapy for insomnia (CBT-I) is recommended as the first-line treatment, access is limited. Acupuncture has demonstrated therapeutic benefits for insomnia but requires trained practitioners and repeated clinic visits. Low-level laser therapy (LLLT), an acupoint-targeted form of photobiomodulation that is also referred to as laser acupuncture when applied to acupuncture points, offers a non-invasive and standardized modality that may serve as a practical alternative.

**Methods:**

This single-center, parallel-group, assessor-blinded, superiority randomized controlled trial will compare wearable LLLT with manual acupuncture in 106 adults with chronic insomnia. Participants aged 18–65 years who meet DSM-5 criteria for chronic insomnia and have an Insomnia Severity Index (ISI) score between 8 and 21 will be randomly allocated (1:1) to the LLLT group or acupuncture group. Both interventions will target bilateral HT7, PC6 and SP6 (six acupoints in total) for 30 minutes per session, three times per week, over two weeks (six sessions total). In the LLLT group, 650-nm laser irradiation will be delivered simultaneously to all six acupoints. The primary outcome is the change in ISI score from baseline to week 2. Secondary outcomes include sleep quality (Pittsburgh Sleep Quality Index, PSQI), fatigue (Fatigue Severity Scale, FSS), depressive symptoms (Patient Health Questionnaire-9, PHQ-9), anxiety (Generalized Anxiety Disorder-7, GAD-7) and heart rate variability (HRV) indices. The ISI will be assessed at baseline, post-treatment (week 2), and follow-up (week 6). Secondary outcomes, including PSQI, FSS, PHQ-9, GAD-7, and HRV indices, will be assessed at baseline and week 2. Analyses will follow the intention-to-treat principle using analysis of covariance and prespecified longitudinal or repeated-measures models where appropriate.

**Discussion:**

This trial will provide comparative evidence on the short-term efficacy and safety of wearable LLLT versus manual acupuncture for chronic insomnia. By directly comparing an established acupuncture protocol with a non-invasive intervention that has potential for future home-based implementation, the study aims to clarify the clinical value of LLLT as a less invasive alternative within the framework of complementary and integrative sleep medicine.

**Clinical trial registration:**

## Highlights

- This randomized, assessor-blinded superiority trial directly compares wearable low-level laser therapy (LLLT) with manual acupuncture for chronic insomnia.
- Both interventions stimulate the same standardized acupoints (HT7, PC6, SP6), allowing a head-to-head comparison of a non-invasive versus invasive protocol.
- The trial evaluates short-term effects on insomnia severity, sleep quality, mood, fatigue, and heart rate variability, with additional four-week follow-up assessment of insomnia severity.

## Introduction

Chronic insomnia affects approximately 10–15% of adults and is associated with significant impairments in quality of life, cognitive performance and mood, as well as increased cardiovascular and metabolic risk. It is characterized by persistent difficulty initiating or maintaining sleep, or early morning awakenings with daytime consequences, and often becomes a chronic and relapsing condition (1).

Cognitive behavioral therapy for insomnia (CBT-I) is widely endorsed as the gold-standard, first-line treatment (2). However, its implementation in real-world settings is constrained by a shortage of trained therapists, time-intensive protocols and limited patient adherence. Pharmacologic treatments such as benzodiazepines and non-benzodiazepine hypnotics may provide short-term relief but are associated with tolerance, dependence and adverse effects, especially in long-term use (2).

Beyond behavioral and pharmacological approaches, cognitive and metacognitive processes (e.g., maladaptive beliefs, arousal and acceptance-related mechanisms) have been implicated in the maintenance of insomnia, supporting interest in interventions that may modulate these pathways (3).

Acupuncture has been increasingly used as a complementary approach for insomnia. Systematic reviews and randomized controlled trials indicate that acupuncture can improve sleep quality and insomnia severity and is generally considered safe when performed by trained practitioners (4–8). Nevertheless, acupuncture has limitations, including invasiveness (needling), requirement for clinic-based sessions and dependence on individual practitioner skill, which can restrict its scalability and patient acceptability (7, 8).

Low-level laser therapy (LLLT) is a form of photobiomodulation that delivers low-power laser light to biological tissues. When applied to acupuncture points, LLLT is also referred to as laser acupuncture. In the present protocol, the term LLLT is used consistently to describe the acupoint-targeted low-power laser intervention. LLLT is thought to modulate cellular and microcirculatory processes, influence autonomic function, and impact neuroendocrine pathways relevant to sleep regulation11. Evidence from laser acupuncture trials and broader photobiomodulation studies suggests potential effects on sleep-related symptoms and neurophysiological processes6 (9–11),. Compared with manual acupuncture, wearable acupoint-targeted LLLT may provide a non-invasive and more standardized modality, with potential advantages in repeatability, acceptability, and future home-based implementation (9–12). Within the photobiomodulation framework, biological effects depend on wavelength, irradiance, fluence, and tissue optical properties; at acupoint sites, 650-nm red light is expected to act primarily on superficial tissues and local neurovascular structures (9–12).

The selected acupoints were chosen because they are commonly used in acupuncture trials and clinical practice for insomnia and related emotional symptoms. HT7 (Shenmen) is commonly selected in acupuncture protocols for sleep disturbance and emotional dysregulation; PC6 (Neiguan) is frequently used in conditions involving autonomic and affective symptoms; and SP6 (Sanyinjiao) is commonly included in protocols for insomnia and stress-related symptoms (4–8). From a neurophysiological perspective, stimulation at these peripheral acupoints may influence local sensory afferents, microcirculatory responses, and autonomic regulation. Therefore, bilateral stimulation of HT7, PC6, and SP6 provides a biologically plausible acupoint combination for evaluating changes in insomnia severity and HRV-related autonomic indices.

Mechanistic neurostimulation evidence at acupoints (e.g., changes in functional networks after acupoint-based stimulation) further supports the plausibility of acupoint-targeted neuromodulation for insomnia-related outcomes (13). To our knowledge, head-to-head randomized trials directly comparing acupoint-targeted LLLT with manual acupuncture for chronic insomnia remain scarce, especially those incorporating HRV as a complementary physiological outcome.

### Rationale for an active-comparator superiority trial

Given the existing evidence supporting acupuncture in insomnia (4–8) and the practical advantages of wearable LLLT (9–12), a key clinical question is whether LLLT can produce a greater improvement in insomnia severity than acupuncture under a prespecified superiority framework. Instead of using a placebo or sham control, this trial adopts an active-comparator design, directly comparing LLLT with an established acupuncture protocol. This approach reflects real-world clinical decision-making, where patients and clinicians may need to choose between different active interventions (7, 8).

Placebo-controlled designs for acupuncture and laser-based interventions present substantial methodological challenges, including the difficulty of creating credible sham procedures. Prior acupuncture and related protocol work illustrates common design choices and feasibility considerations relevant to the current schedule and outcomes (14). By choosing manual acupuncture as the comparator, this trial aims to provide clinically meaningful evidence on whether LLLT can serve as a practical, non-invasive alternative to a widely accepted complementary therapy (7, 8, 12).

### Objectives

The primary objective of this study is to evaluate whether wearable LLLT produces a greater reduction in insomnia severity than manual acupuncture after a two-week treatment course in adults with chronic insomnia.

Secondary objectives are to compare the effects of LLLT and acupuncture on sleep quality, fatigue, depressive and anxiety symptoms and autonomic function assessed by heart rate variability.

### Primary hypothesis

We hypothesize that, compared with acupuncture, LLLT will result in a greater reduction in ISI scores from baseline to the end of the two-week intervention period (superiority hypothesis). Although comparative evidence remains limited, the superiority hypothesis was specified *a priori* on the basis of the pilot findings and the practical advantages of a standardized, non-invasive acupoint-based intervention.

## Methods

### Trial design and setting

**Figure 1:**
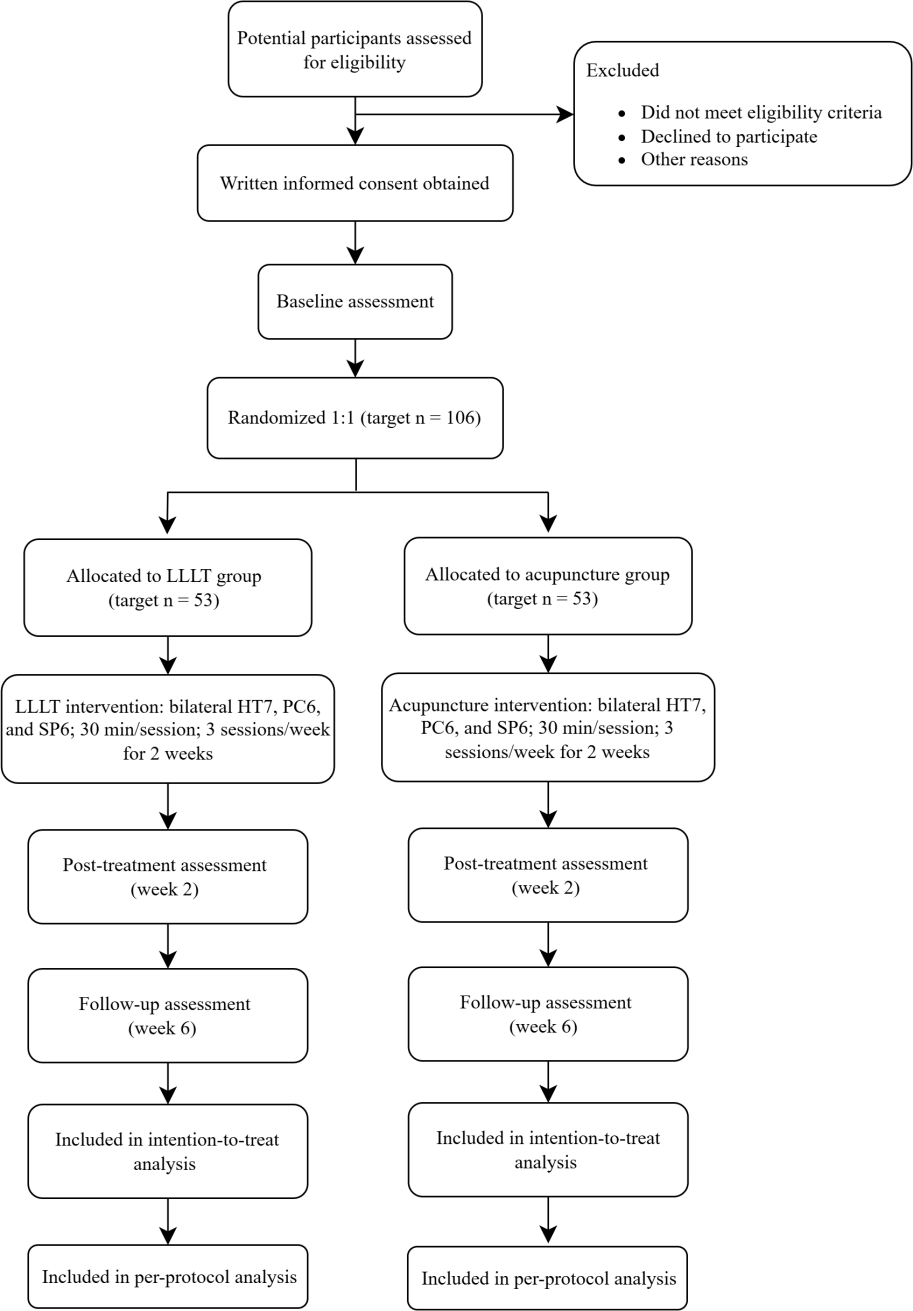
Planned participant flow of the trial. Potential participants with chronic insomnia will be screened according to the eligibility criteria, undergo baseline assessment and written informed consent, and then be randomized in a 1:1 ratio to the LLLT group or the acupuncture group. Both groups will receive 2 weeks of intervention, followed by post-treatment assessments at week 2 and an ISI follow-up assessment at week 6. LLLT, low-level laser therapy; ISI, Insomnia Severity Index.

This protocol describes a single-center, parallel-group, assessor-blinded, superiority randomized controlled trial conducted at Guangdong Provincial Hospital of Traditional Chinese Medicine. The trial has been approved by the institutional ethics committee and was prospectively registered (ITMCTR2025000977) (15). The protocol follows the Standard Protocol Items: Recommendations for Interventional Trials (SPIRIT) (16) and will be reported in accordance with the CONSORT (17) and STRICTA statements (18). The overall study flow, including enrolment, allocation, follow-up, and analysis, is summarized in **Figure 1**.

### Participants

Adults with chronic insomnia will be recruited from outpatient clinics and hospital advertisements.

#### Inclusion criteria

- Age 18–65 years, either sex;
- Diagnosis of chronic insomnia according to DSM-5 (19);
- Insomnia Severity Index (ISI) total score ≥ 8 and < 22 (mild to moderate insomnia) (20, 21);
- Insomnia duration ≥ 3 months;
- Able and willing to provide written informed consent.

#### Exclusion criteria

- Severe insomnia (ISI ≥ 22) (20, 21);
- Major psychiatric disorders (e.g. major depression, psychosis) or high scores on PHQ-9 (≥ 10) (22) or GAD-7 (≥ 10) (23);
- Secondary insomnia due to other primary sleep or medical disorders (e.g. sleep apnea, uncontrolled cardiovascular disease);
- Recent use of hypnotics or structured CBT-I within the previous 3 months;
- Contraindications to LLLT or acupuncture (photosensitivity, bleeding tendency, needle phobia, skin lesions at acupoints);
- Pregnancy or lactation;
- Participation in another clinical trial within the previous 3 months.

### Recruitment and informed consent

Participants will be recruited through hospital outpatient clinics, posters and flyers in the hospital, institutional websites and social media platforms. Potential participants will undergo initial screening to assess eligibility. Those who meet preliminary criteria will be invited for baseline assessment and detailed explanation of the study procedures. Written informed consent will be obtained from all participants before any study-specific procedures are conducted.

### Randomization and allocation concealment

Participants will be randomized to the LLLT or acupuncture group in a 1:1 ratio using a computer-generated randomization sequence created by an independent statistician using SPSS (version 26.0). Randomization will be stratified according to baseline insomnia severity (mild: ISI 8–14; moderate: ISI 15–21) to ensure balanced distribution across groups. Allocation concealment will be maintained using sequentially numbered, opaque, sealed envelopes prepared and safeguarded by the independent statistician. After baseline assessments and confirmation of eligibility, the study coordinator will open the next envelope in sequence to assign the participant to a treatment group. The statistician responsible for generating and guarding the randomization list will have no involvement in clinical assessments, intervention delivery, or outcome analysis.

### Blinding

Due to the nature of the interventions, participants and treating practitioners cannot be blinded to group allocation. However, outcome assessors, including those administering questionnaires and conducting HRV measurements, will be blinded to treatment allocation. Data managers and statisticians performing the analyses will also remain blinded to group assignments. Participants will be instructed not to disclose their treatment allocation to assessors, and any accidental unblinding of assessors will be documented with the reason recorded.

We acknowledge, however, that because the primary outcome and most secondary outcomes are patient-reported, assessor blinding cannot eliminate performance bias or reporting bias arising from participants’ awareness of treatment allocation. This limitation will therefore be explicitly considered in the interpretation of the findings.

### Interventions

**Figure 2:**
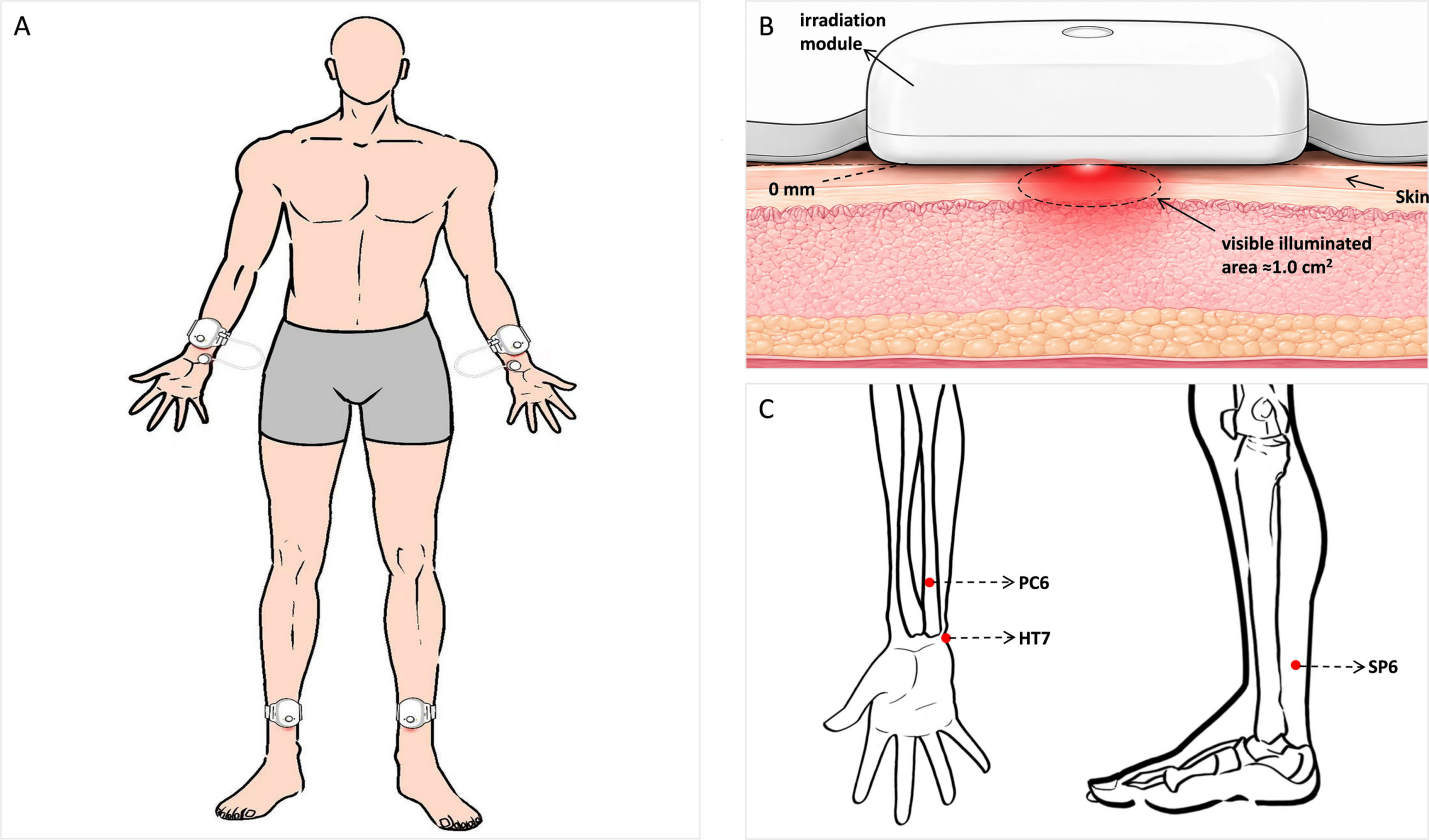
Wearable LLLT irradiation setup, skin-contact configuration, and acupoint positioning. **(A)** Overall placement of the wearable irradiation setup, including wrist-mounted main units, external single-point irradiation probes, and lower-leg modules used for simultaneous bilateral stimulation of HT7, PC6, and SP6. This panel is intended to illustrate the overall device placement. **(B)** Schematic illustration of the skin-contact irradiation configuration. The irradiation module was placed in direct contact with the skin surface, corresponding to an irradiation distance of 0 mm. Although the physical laser outlet was small, the visible illuminated area at the skin-contact surface during device activation was approximately 1.0 cm². The 650-nm working mode was used, with a manufacturer-reported terminal output power of ≤5 mW per irradiation outlet. Dose estimates were calculated at the level of each acupoint, and the total session energy represents the summed exposure across six anatomically separate treated sites. **(C)** Standardized anatomical localization of HT7, PC6, and SP6 used for bilateral irradiation. Panels **(B, C)** should be considered the primary references for reproducibility, whereas the overall device depiction in Panel **(A)** is provided for illustrative purposes. The schematic is not to scale.

Both groups will receive interventions targeting the same standardized acupoints—HT7 (Shenmen), PC6 (Neiguan), and SP6 (Sanyinjiao)—applied bilaterally, corresponding to six acupoints in total. These acupoints are frequently used in insomnia-related acupuncture research and are considered relevant to sleep regulation, emotional symptoms, and autonomic modulation in previous clinical studies (4–8). Session duration will be 30 minutes, three times per week for two weeks, for a total of six sessions. The wearable LLLT irradiation setup, skin-contact configuration, and standardized anatomical locations of the selected acupoints are shown in **Figures 2A–C**. Technical and dosimetric parameters of the LLLT intervention are summarized in **Table 1**. Needling details for the acupuncture group are summarized in **Table 2**, and the assessment schedule is presented in **Table 3**. Standardized operating procedures developed specifically for this trial will be used to ensure consistency across sessions and participants.

**Table 1:** Technical and dosimetric parameters of the LLLT intervention.

Parameter	Description
Device name	Wearable dual-spectrum meridian-regulation research prototype
Model	BW-SH23-2
Manufacturer	Guangzhou XinXin Medical Co., Ltd., China
Available wavelengths	650 ± 10 nm and 808 ± 10 nm
Wavelength used in this trial	650 nm only
Working mode	Built-in 650-nm working mode
Output power	Manufacturer-reported maximum terminal output power at 650 nm: ≤5 mW per irradiation outlet
CW/pulsed status, pulse frequency, and duty cycle	Not specified in the manufacturer’s documentation; no independent temporal waveform measurement was performed.
Treatment sites	Bilateral HT7, PC6, and SP6
Number of treated sites	Six acupoints
Irradiation sequence	Simultaneous irradiation
Contact condition	Direct contact with skin
Irradiation distance	0 mm
Skin-contact illuminated area	Approximately 1.0 cm², estimated from the visible illuminated area at the skin-contact surface during device activation
Estimated irradiance	Approximately 5 mW/cm²
Session duration	30 minutes
Estimated energy per acupoint	Approximately 9 J
Estimated fluence per acupoint	Approximately 9 J/cm²
Estimated total energy per session	Approximately 54 J across six acupoints
Fixation method	Adjustable elastic straps and adhesive single-point irradiation modules
Laser safety classification	Class 3B
Safety precautions	Avoid direct ocular exposure and direct viewing of the laser window; switch off the device before module removal or repositioning; monitor skin irritation, discomfort, dizziness, visual discomfort, and other adverse events

LLLT, low-level laser therapy. Irradiance, fluence, and total energy values are protocol-level estimates based on manufacturer-reported specifications, the assumed maximum terminal output power at 650 nm, the observed visible illuminated area at the skin-contact surface, and the planned irradiation duration. These values were not obtained from independent optical calibration, beam-profile assessment, or spatial power-distribution mapping. The manufacturer’s documentation did not specify whether the 650-nm output was continuous-wave or pulsed, nor did it provide pulse frequency or duty-cycle parameters. The estimated total energy per session represents the summed exposure across six anatomically separate acupoints and should not be interpreted as the local dose delivered to a single acupoint.

**Table 2:** Acupuncture location and needling method for each acupoint.

Acupoint	Location	Needling method
Shenmen (HT7)	On the palmar ulnar end of the transverse crease of the wrist, at the radial side of the tendon of the flexor carpi ulnaris.	Puncture perpendicularly 0.5–1 cun.
Neiguan (PC6)	On the line joining Daling and Quze, between the tendons of palmaris longus and flexor carpi radialis, 2 cun above the wrist crease.	Puncture perpendicularly 1–1.5 cun.
Sanyinjiao (SP6)	Posterior to the medial border of the tibia and 3 cun above the tip of the medial malleolus.	Puncture perpendicularly 1–1.5 cun.

Locations and needling methods follow the WHO Standard Acupuncture Point Locations in the Western Pacific Region. The deqi sensation will be elicited using manual manipulation.

**Table 3:** Schedule of enrolment, interventions, and assessments.

Item	Week 0 baseline	Week 1 treatment	Week 2 end of treatment	Week 3 follow-up	Week 4 follow-up	Week 5 follow-up	Week 6 follow-up
Enrolment and allocation							
Telephone pre-screening/reservation	×						
Eligibility screening/enrolment	×						
Written informed consent	×						
Randomization/allocation	×						
Physical examination/safety check	×		×				
Interventions							
LLLT group		3 sessions	3 sessions				
Manual acupuncture group		3 sessions	3 sessions				
Outcome assessments							
ISI	×		×				×
PSQI	×		×				
FSS	×		×				
PHQ-9	×		×				
GAD-7	×		×				
HRV	×		×				
Process and safety monitoring							
Sleep diary	×	×	×	×	×	×	×
Adverse event monitoring		×	×	×	×	×	×
Concomitant medication recording	×	×	×	×	×	×	×
Dropouts/withdrawals		×	×	×	×	×	×
Participant adherence		×	×				

× indicates that the item is performed or assessed at the corresponding time point. LLLT, low-level laser therapy; ISI, Insomnia Severity Index; PSQI, Pittsburgh Sleep Quality Index; FSS, Fatigue Severity Scale; PHQ-9, Patient Health Questionnaire-9; GAD-7, Generalized Anxiety Disorder-7; HRV, heart rate variability. Week 0 indicates baseline, week 2 marks the end of treatment, and week 6 represents follow-up. Both interventions are delivered three times per week for 2 weeks, with six sessions in total. ISI is assessed at baseline, week 2, and week 6; PSQI, FSS, PHQ-9, GAD-7, and HRV are assessed at baseline and week 2. Sleep diaries, adverse events, concomitant medications, dropouts/withdrawals, and adherence are monitored as indicated.

#### LLLT group

Participants allocated to the LLLT group will receive treatment using a wearable dual-spectrum meridian-regulation research prototype (model BW-SH23-2; Guangzhou XinXin Medical Co., Ltd., China). According to the manufacturer’s instructions, the device supports laser outputs at 650 ± 10 nm and 808 ± 10 nm. In the present trial, only the built-in 650-nm working mode will be used.

The manufacturer-reported maximum terminal output power at 650 nm is ≤5 mW per irradiation outlet. The device provides selectable wavelength modes, including 650 nm, 808 nm, and combined 650/808 nm modes; however, only the built-in 650-nm working mode will be used in this trial. According to the manufacturer’s specifications, the temporal emission mode of the 650-nm output is not specified; therefore, it is unclear whether the output is continuous-wave or pulsed. Pulse frequency and duty-cycle parameters are also not provided. No independent optical calibration, temporal waveform measurement, beam-profile assessment, or spatial power-distribution mapping was performed for this protocol. This absence of independent temporal and spatial optical characterization represents a methodological limitation and may limit direct comparability with photobiomodulation studies that report calibrated optical output, pulse structure, and beam-profile characteristics. Accordingly, all reported optical and dosimetric values should be interpreted as protocol-level estimates based on manufacturer-reported specifications, the planned irradiation duration, and the observed visible illuminated area at the skin-contact surface. In the context of this protocol study, this level of reporting was considered acceptable because the aim was to provide a transparent and reproducible description of the planned intervention using all device information available before trial implementation.

In the present trial, one irradiation outlet/module was positioned over each target acupoint, corresponding to six 650-nm irradiation outlets used simultaneously for bilateral HT7, PC6, and SP6. Each outlet was aligned with a single acupoint, and the manufacturer-reported terminal output power was ≤5 mW for each 650-nm outlet. Thus, the estimated local dose was calculated at the level of each individual acupoint, while the total session energy was calculated as the sum of the six anatomically separate irradiation sites.

Before each treatment session, bilateral HT7, PC6, and SP6 will be identified according to standardized anatomical landmarks by trained research staff. After skin cleaning, each irradiation module will be aligned with the corresponding acupoint and applied in direct contact with the skin surface, with an irradiation distance of 0 mm. Although the physical laser outlet of each irradiation module is small, the visible illuminated area at the skin-contact surface during device activation is approximately 1.0 cm², as estimated from the observed light spot. The irradiation modules will be secured using adjustable elastic straps and adhesive single-point modules to improve positioning consistency across sessions.

Each treatment session will last 30 minutes. Assuming the maximum manufacturer-reported terminal output power of 5 mW at 650 nm and an estimated visible illuminated skin-contact area of approximately 1.0 cm², the estimated maximum irradiance is approximately 5 mW/cm². For each 30-minute session, the maximum delivered energy is estimated to be 9 J per acupoint, corresponding to an estimated fluence of approximately 9 J/cm² per acupoint. Because irradiation is delivered simultaneously to six anatomically separate acupoints, the estimated total delivered energy is approximately 54 J per session across all treated sites. This total session energy represents the summed exposure across the six treated acupoints and should not be interpreted as the local dose delivered to a single acupoint. These dosimetric values are protocol-level estimates based on manufacturer-reported output power and the observed visible illuminated area at the skin-contact surface, rather than direct measurements obtained from optical power mapping. Because six acupoints are irradiated simultaneously, potential cumulative or interaction effects across treated sites cannot be fully excluded. This possibility will be considered when interpreting clinical and physiological outcomes.

Basic laser safety procedures will be followed throughout treatment. The device is classified in the manufacturer’s instructions as a Class 3B laser device. Direct ocular exposure to the laser window will be strictly avoided. During operation, the irradiation outlet will be aligned with the target acupoint and placed in direct contact with the skin before activation whenever feasible, and the device will be switched off before module removal or repositioning. Direct viewing of the laser window will be prohibited throughout the procedure. Participants will be monitored for local skin irritation, discomfort, dizziness, visual discomfort, or other adverse events during and after treatment.

#### Acupuncture group

Participants in the acupuncture group will be treated by licensed acupuncturists with at least five years of clinical experience. All practitioners will undergo dedicated training on the study protocol and operating procedures before the start of the trial.

Acupoints: bilateral HT7 (Shenmen), PC6 (Neiguan) and SP6 (Sanyinjiao). Acupoint locations will follow the WHO Standard (24) Acupuncture Point Locations in the Western Pacific Region. Sterile, single-use stainless-steel needles (0.25 mm × 25 mm) will be used. After skin disinfection, needles will be inserted perpendicularly at each acupoint (HT7: 0.5–1 cun; PC6: 1–1.5 cun; SP6: 1–1.5 cun). Manual stimulation (twirling, lifting and thrusting) will be applied to elicit the deqi sensation. Needles will be retained for 30 minutes, with additional mild stimulation every 10–15 minutes if needed.

### Treatment fidelity and adherence

To promote intervention fidelity, all practitioners and staff will receive standardized training before the trial. To improve positioning reproducibility across sessions, bilateral HT7, PC6, and SP6 will be re-identified at each treatment session using standardized anatomical landmarks by the same trained research personnel whenever possible. The irradiation outlets will be aligned with the marked acupoint sites before activation, and direct skin contact will be maintained throughout the session. Adjustable elastic straps and adhesive single-point irradiation modules will be used to minimize displacement and variability in positioning. The position and contact condition of each module will be checked at the beginning of treatment and, when necessary, during the session to maintain consistent alignment and skin-contact conditions across sessions. Checklists will be used during sessions to record adherence to operating procedures. Attendance at each treatment session will be recorded. Participant adherence will be monitored using attendance logs and brief compliance interviews, and reasons for missed sessions will be documented.

### Outcomes

#### Primary outcome

The primary outcome is the change in Insomnia Severity Index (ISI) total score from baseline to the end of the two-week intervention (week 2). The ISI is a 7-item self-report scale that assesses the severity of nocturnal and daytime components of insomnia over the past two weeks, with total scores ranging from 0 to 28. Higher scores indicate more severe insomnia. The ISI has been validated in Chinese populations and demonstrates good reliability and sensitivity to change (20, 21).

#### Secondary outcomes

- Pittsburgh Sleep Quality Index (PSQI) to assess subjective sleep quality over the past month; because the PSQI recall window is longer than the 2-week intervention period, the week-2 PSQI analysis will be interpreted cautiously as a supportive secondary endpoint (25);
- Fatigue Severity Scale (FSS) to evaluate the impact and severity of fatigue on daily functioning (26);
- Patient Health Questionnaire-9 (PHQ-9) to assess depressive symptoms;
- Generalized Anxiety Disorder-7 (GAD-7) to assess anxiety symptoms;
- Heart rate variability (HRV) parameters, including SDNN, RMSSD, LF, HF, and LF/HF ratio, will be measured using the SA-3000P Mental Stress Analysis System (Medicore, Republic of Korea) under standardized short-term resting conditions with a finger-clip photoplethysmography (PPG) sensor to assess changes in autonomic regulation before and after the intervention. HRV is included as an exploratory physiological outcome and will be interpreted as reflecting autonomic regulation rather than sleep architecture directly (27–30).

#### HRV measurement procedure

**Figure 3:**
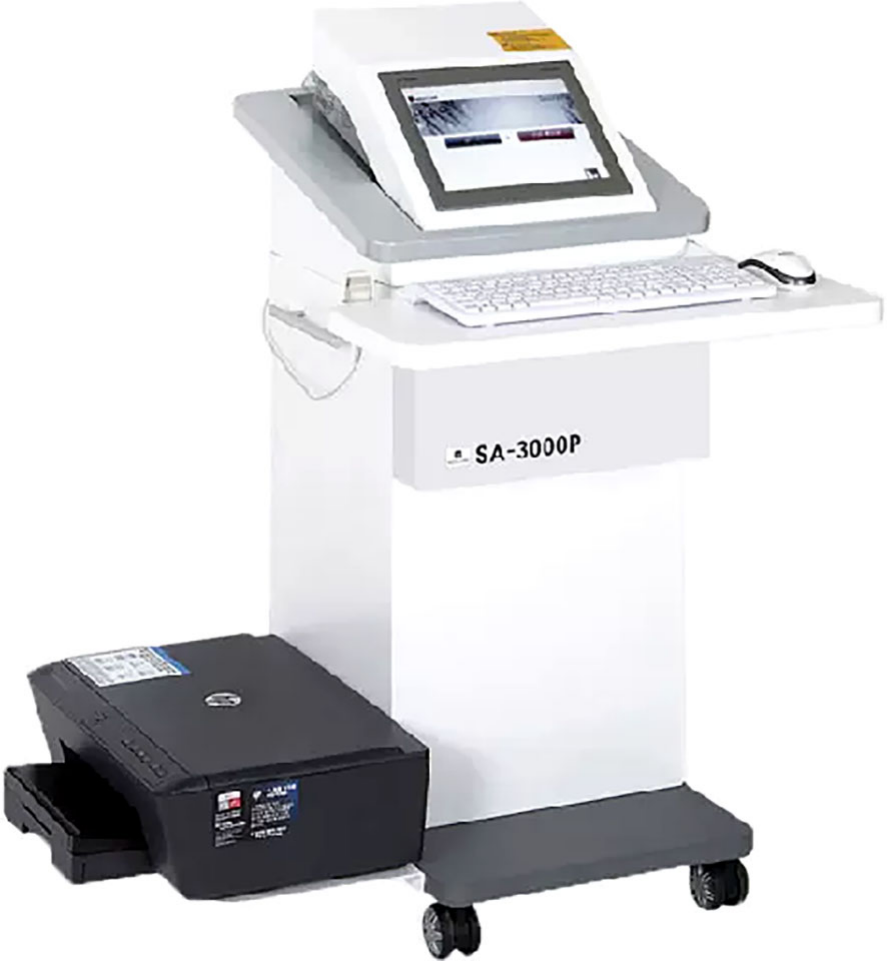
SA-3000P system and sensor setup used for short-term resting HRV assessment. Heart rate variability (HRV) in this study is assessed using the SA-3000P Mental Stress Analysis System (Medicore, Republic of Korea) with a finger-clip photoplethysmography (PPG) sensor. The device is used for standardized 5-minute resting recordings under controlled conditions to obtain time-domain and frequency-domain HRV indices, including SDNN, RMSSD, LF, HF, and LF/HF ratio. HRV, heart rate variability; PPG, photoplethysmography; SDNN, standard deviation of normal-to-normal intervals; RMSSD, root mean square of successive differences; LF, low frequency; HF, high frequency.

HRV assessments will be performed using the SA-3000P Mental Stress Analysis System (Medicore, Republic of Korea), an HRV analysis platform equipped with both electrocardiography (ECG) and photoplethysmography (PPG) acquisition modalities. In the present study, short-term resting HRV will be obtained using the dedicated finger-clip PPG sensor supplied with the system. The device derives inter-beat interval (IBI) series from peripheral pulse-wave signals and provides time-domain and frequency-domain HRV indices, including SDNN, RMSSD, LF, HF, and LF/HF ratio. The HRV measurement device and sensor setup used in this study are shown in **Figure 3**.

Assessments will be conducted in a dedicated quiet room with the ambient temperature maintained at 23–25°C. After entering the room, participants will sit comfortably with both hands naturally relaxed on the thighs or desk and rest quietly for 5–10 minutes before data acquisition in order to reduce the influence of recent movement and transient emotional fluctuations on autonomic activity. Participants will be instructed to breathe naturally and steadily, to avoid intentional deep breathing or breath holding, and to refrain from talking or unnecessary body or finger movements during the recording period.

For signal acquisition, the finger-clip PPG sensor will be placed on the participant’s left index finger. Before measurement, the fingertip will be checked to ensure that the skin is clean and dry and that there is no obvious contamination, nail polish, or excessive nail length that might interfere with optical signal acquisition. Before formal recording, waveform preview and signal-quality checking will be performed. If the waveform is weak, irregular, or obviously affected by interference, the clip position will be adjusted and the connection rechecked until a clear and stable waveform is obtained. Once the signal is stable and artifact interference is minimal, 5 minutes of continuous resting data will be recorded for HRV analysis.

To improve data reliability, several quality-control procedures will be applied. First, during the recording period, participants will remain relaxed and quiet and will avoid talking, coughing, exaggerated swallowing, or finger movement; if obvious waveform distortion occurs, the measurement will be paused and repeated. Second, participants will be instructed to avoid alcohol intake and sleep deprivation within 24 hours before testing and to avoid strong tea, coffee, and vigorous exercise within 2 hours before testing. Third, participants with obvious arrhythmias that materially impair HRV interpretation, such as atrial fibrillation or frequent premature beats, will be excluded from HRV statistical analyses. Finally, all HRV measurements will be performed by the same trained researcher using the same device and under as consistent conditions as possible, including room environment, body position, pre-test rest period, recording duration, and approximate time of day.

Because the PPG-based approach provides pulse-to-pulse intervals as a surrogate for cardiac beat-to-beat intervals, HRV in this study will be interpreted as an exploratory physiological indicator of autonomic regulation rather than a direct measure of sleep architecture or sleep continuity. Accordingly, HRV findings will be interpreted with appropriate caution in light of measurement timing, peripheral perfusion conditions, and inter-individual heterogeneity.

#### Safety outcomes

Adverse events related to LLLT or acupuncture, such as skin irritation, transient dizziness, bleeding, hematoma or pain, will be monitored at each visit and recorded in standardized forms. Severity, duration and causal relationship with the intervention will be assessed and documented.

### Participant timeline

Study procedures will take place at baseline (week 0), during the treatment phase, at the end of treatment (week 2), and at follow-up (week 6). Baseline assessments include demographic data, medical history, physical examination, ISI, PSQI, FSS, PHQ-9, GAD-7, and HRV. At week 2, ISI, PSQI, FSS, PHQ-9, GAD-7, and HRV will be reassessed, and a brief physical examination or safety check will also be repeated to support adverse-event monitoring. At week 6, only ISI will be reassessed to evaluate the persistence of the primary insomnia-severity outcome. Sleep diaries, adverse events, and concomitant medications will be monitored throughout the study as specified in **Table 3**.

### Sample size calculation

The sample size was formally powered based on the primary outcome, which is the detection of a clinically and statistically significant difference in ISI scores between the two groups. In our single-arm pilot study of wearable LLLT involving 33 participants, the baseline ISI score was 15.12 ± 3.72 and the post-intervention ISI score after the 2-week intervention was 10.91 ± 4.44, corresponding to a mean reduction of 4.21 points. For the manual acupuncture comparator, a randomized controlled trial reported a mean ISI score of 13.4 ± 3.9 in the acupuncture group at 2 weeks post-treatment (baseline 17.1 ± 4.1)5. Because directly comparable head-to-head change-score data for wearable LLLT versus manual acupuncture were unavailable, the sample size calculation was based on post-treatment ISI means from our pilot LLLT data and a published acupuncture trial. This approach was used as a pragmatic estimate for the expected between-group difference at the primary endpoint, while the primary analysis will adjust for baseline ISI using ANCOVA. To establish superiority, we assumed a conservative between-group difference of 2.5 points in favor of the LLLT group. Using a two-sample t-test allowing unequal variances in PASS software (version 21.0.3), with a two-sided significance level of 0.05 and 80% power, the required sample size was calculated to be 90 participants, with 45 participants per group. Allowing for an anticipated 15% dropout rate to maintain statistical power, the final target sample size was set at 106 participants, with 53 participants per group.

### Statistical analysis

All analyses will be performed using SPSS (version 26.0) or equivalent software. Two-tailed p-values < 0.05 will be considered statistically significant. The primary analysis will strictly follow the intention-to-treat (ITT) principle. For the primary superiority outcome, analysis of covariance (ANCOVA) will be employed. Although the primary endpoint is defined as the change in ISI from baseline to week 2, ANCOVA will be performed with the week-2 ISI score serving as the dependent variable, the treatment allocation (LLLT vs. acupuncture) as the fixed factor, and the baseline ISI score as a covariate (31). This approach is statistically equivalent to comparing change scores while providing better precision. Furthermore, this adjustment for baseline ISI is specifically chosen to align with the stratified block randomization procedure, thereby maximizing statistical power and precision in estimating the treatment effect.

Between-group differences in adjusted mean changes and their 95% confidence intervals (CIs) will be reported. Statistical superiority of LLLT over manual acupuncture for the primary endpoint will be considered supported if the two-sided p-value for the between-group difference is less than 0.05 and the entire 95% CI of the difference excludes zero in favor of the LLLT group (32).

The longitudinal trajectory of ISI across post-randomization assessments will be analyzed as a supportive repeated-measures analysis using a linear mixed-effects model. The model will include treatment group, time, and the group × time interaction as fixed effects, baseline ISI as a covariate, and participant-specific random intercepts to account for within-subject correlations. This model will be used to evaluate whether the between-group difference in insomnia severity is maintained at week 6.

Secondary continuous outcomes assessed at week 2 only, including PSQI, FSS, PHQ-9, GAD-7, and HRV indices, will be analyzed using analysis of covariance, with the week-2 value as the dependent variable, treatment group as the fixed factor, and the corresponding baseline value as a covariate. When model assumptions are markedly violated, appropriate transformations or non-parametric methods will be considered. A per-protocol analysis including participants with adequate adherence, defined as attending at least four of six sessions and having no major protocol deviations, will be performed as a sensitivity analysis.

For the primary ANCOVA analysis, missing week-2 ISI data will be handled using multiple imputation under the missing-at-random assumption. The imputation model will include baseline characteristics, treatment allocation, and available outcome data, and results from imputed datasets will be combined using Rubin’s rules (33). For the supportive longitudinal ISI analysis, the linear mixed-effects model will use all available ISI observations under the same missing-at-random assumption. For secondary outcomes assessed at week 2 only, missing data will be handled using multiple imputation if the extent of missingness is substantial, and complete-case or imputation-based sensitivity analyses will be conducted as appropriate.

### Data collection and management

Data will be collected using standardized case report forms by trained research personnel. Each participant will be assigned a unique study identification number to ensure confidentiality. Data will be entered into a secure, password-protected electronic database by two independent data entry staff members (double data entry). Discrepancies will be resolved by referring to the original records. The database will be backed up regularly on encrypted institutional servers, and access will be restricted to authorized personnel.

### Adverse events and safety monitoring

All adverse events will be recorded throughout the study, with details on onset, duration, severity and their relationship to the intervention. Serious adverse events will be reported promptly to the ethics committee in accordance with institutional requirements. Participants experiencing significant adverse events may discontinue the study at the discretion of the investigator.

### Patient and public involvement

Patients or members of the public were not involved in the design, conduct, reporting or dissemination plans of this trial.

### Researchers training

Before participant enrolment, all investigators (acupuncturists, LLLT operators, assessors and data managers) will receive standardized training on the study protocol, operating procedures and data collection forms in order to ensure consistency across visits.

## Discussion

This protocol describes a randomized, assessor-blinded, superiority randomized controlled trial designed to compare wearable low-level laser therapy (LLLT) with manual acupuncture for the treatment of chronic insomnia. Acupuncture has accumulated evidence as an effective complementary therapy for insomnia (4–8), but its invasiveness, requirement for clinic-based treatment and dependence on practitioner skill may limit its accessibility and scalability. Wearable LLLT, applied to standardized acupoints, offers a non-invasive intervention with potential for future home-based implementation (9–12). By directly comparing these two active interventions that stimulate the same bilateral acupoints (HT7, PC6, and SP6), this trial aims to provide clinically meaningful evidence on whether LLLT produces greater improvement in insomnia severity than manual acupuncture.

Compared with previous trials, this study has several methodological strengths. First, it adopts a parallel-group randomized design with allocation concealment and blinded outcome assessment where feasible. This approach helps reduce selection bias and assessor-related bias for investigator-administered procedures such as HRV measurement, although it cannot eliminate expectation-related bias in patient-reported outcomes. Second, both groups receive rigorously standardized interventions, including clearly defined acupoint locations, treatment frequency and session duration, thereby enhancing reproducibility. Third, the trial incorporates both subjective and physiological outcome measures: the ISI and PSQI capture patients’ perceived insomnia severity and sleep quality, while HRV indices provide complementary information on autonomic regulation that may be associated with treatment-related changes. In addition, the use of validated questionnaires for fatigue, depressive and anxiety symptoms allows a broader evaluation of comorbid symptoms that are highly relevant in chronic insomnia.

The statistical analysis plan is also a strength of this protocol. The sample size is based on preliminary data and previous acupuncture trials, and the primary analysis follows a superiority framework using ANCOVA to adjust for baseline differences. The week-6 ISI follow-up will be examined using a supportive linear mixed-effects model, while secondary continuous outcomes assessed at week 2 will be analyzed using ANCOVA with adjustment for the corresponding baseline values. The use of both intention-to-treat and per-protocol analyses, together with prespecified missing-data handling, is expected to provide robust and transparent results.

Nevertheless, several limitations should be acknowledged. First, participants and treating practitioners cannot be blinded, and the primary endpoint together with most secondary endpoints is patient-reported. Accordingly, assessor blinding cannot fully protect the study from expectation effects, performance bias, or reporting bias. Second, the intervention period is relatively short (two weeks), although this schedule was informed by previous acupuncture studies and our pilot data. Longer treatment courses or repeated cycles might yield different or more sustained effects and should be evaluated in future studies. Third, the study is conducted at a single center within a traditional Chinese medicine hospital, which may limit the generalizability of the findings to other settings or healthcare systems. Fourth, the PSQI assesses sleep quality over the preceding month, which means that the week-2 PSQI analysis may not fully reflect within-treatment change over a 2-week intervention and should therefore be interpreted cautiously. Several device-characterization limitations should also be acknowledged. The temporal emission mode of the 650-nm output was not specified in the manufacturer’s documentation; therefore, whether the output is continuous-wave or pulsed remains unclear. In addition, no independent optical calibration, temporal waveform measurement, beam-profile assessment, or spatial power-distribution mapping was performed. As a result, the reported irradiance, fluence, and total energy values should be interpreted as protocol-level estimates based on manufacturer-reported specifications, the planned irradiation duration, and the observed visible illuminated area at the skin-contact surface, rather than directly measured optical parameters. This methodological limitation may restrict comparability with other photobiomodulation studies that report calibrated optical power, pulse structure, beam profile, and spatial power distribution. Nevertheless, because this is a protocol study, transparent reporting of the manufacturer-reported specifications, contact condition, estimated illuminated area, irradiation duration, and calculation assumptions provides a reproducible description of the planned intervention. Future studies should include independent optical calibration, temporal emission characterization, beam-profile assessment, and spatial power-distribution mapping to enable more precise dosimetric reporting. In addition, because six acupoints are irradiated simultaneously, potential cumulative or interaction effects across treated sites cannot be fully excluded and should be considered when interpreting clinical and physiological outcomes.

HRV is included in this trial as an exploratory physiological outcome reflecting autonomic regulation. However, in the present study HRV is derived from short-term resting PPG-based inter-beat interval recordings rather than from polysomnography, actigraphy, or full ECG-based sleep monitoring. Therefore, HRV should not be interpreted as a direct measure of sleep architecture or sleep continuity. We attempted to improve comparability by standardizing posture, room conditions, pre-test rest duration, recording duration, and pre-assessment behavioral restrictions. Nevertheless, peripheral perfusion, minor movement artifacts, time-of-day variation, and participant heterogeneity may still influence HRV estimates and should be considered when interpreting these findings. In addition, chronic insomnia is heterogeneous, and potential contributing factors such as hormonal, metabolic, or other medical drivers are not comprehensively characterized in this protocol, which may contribute to variability in treatment response.

In summary, this superiority randomized controlled trial will systematically evaluate the short-term effects of wearable LLLT compared with manual acupuncture in adults with chronic insomnia, with additional assessment of whether changes in insomnia severity are maintained at four-week follow-up. The findings are expected to inform clinicians and patients about the potential role of LLLT as a practical, non-invasive option within complementary and integrative sleep medicine. Regardless of whether the results demonstrate superiority or no meaningful difference between interventions, the trial will contribute valuable evidence to guide future research and clinical decision-making for chronic insomnia.

### Ethics and dissemination

The study protocol has been reviewed and approved by the Ethics Committee of Guangdong Provincial Hospital of Traditional Chinese Medicine (Approval No. YF2025-141-01). The trial will be conducted in accordance with the Declaration of Helsinki and relevant national regulations. Written informed consent will be obtained from all participants prior to enrolment. Participants may withdraw from the study at any time without prejudice to their routine care (34).

The results of this trial will be disseminated through publication in peer-reviewed journals and presentations at national and international conferences, regardless of whether the findings are positive, negative or inconclusive. De-identified data may be made available upon reasonable request after publication, subject to ethical approval and data-sharing agreements.

### Trial status

Protocol version: 1.3 (July 2025). Participant recruitment began in May 2025 and has been completed. Follow-up assessments and data management are ongoing at the time of manuscript revision. No comparative outcome analysis has been conducted, and treatment allocation remains blinded to outcome assessors and statisticians.
