# Supplementary material for: Wearable low-level laser therapy (laser acupuncture) versus manual acupuncture for chronic insomnia: protocol for a randomized, assessor-blinded, superiority trial
Source: Front Psychiatry. 2026 Jun 1;17:1814245. doi: 10.3389/fpsyt.2026.1814245 (PMC13265499; doi:10.3389/fpsyt.2026.1814245)
Supplement: Supplementary file 1 [file Table1.docx]

**Pilot Study on the Effect of LLLT on ISI Scores (n=33)**

**Study Description:**

A single-arm pre-post trial of LLLT for insomnia was conducted in 33 participants.

ISI scores were measured before and after the 2-week intervention.

SPSS (v27) was used for statistical analysis.

**Descriptive Statistics:**

Baseline ISI: Mean = 15.12, SD = 3.72

Post-intervention ISI: Mean = 10.91, SD = 4.44

Mean reduction = 4.21 points

**Results from SPSS Paired T-Test:**

Paired t-test: t = 5.696, df = 32, p < 0.001

95% CI of mean difference: [2.706, 5.718]

T-检验

| **配对样本统计** | | | | | |
| --- | --- | --- | --- | --- | --- |
|  | | 平均值 | 个案数 | 标准 偏差 | 标准 误差平均值 |
| 配对 1 | 基线_ISI | 15.12 | 33 | 3.723 | .648 |
|  | 干预后_ISI | 10.91 | 33 | 4.440 | .773 |

| **配对样本相关性** | | | | |
| --- | --- | --- | --- | --- |
|  | | 个案数 | 相关性 | 显著性 |
| 配对 1 | 基线_ISI & 干预后_ISI | 33 | .470 | .006 |

| **配对样本检验** | | | | | | | | | |
| --- | --- | --- | --- | --- | --- | --- | --- | --- | --- |
|  | | 配对差值 | | | | | t | 自由度 | Sig.（双尾） |
|  |  | 平均值 | 标准 偏差 | 标准 误差平均值 | 差值 95% 置信区间 | |  |  |  |
|  |  |  |  |  | 下限 | 上限 |  |  |  |
| 配对 1 | 基线_ISI - 干预后_ISI | 4.212 | 4.248 | .740 | 2.706 | 5.718 | 5.696 | 32 | .000 |
