# Supplementary material for: Wearable low-level laser therapy (laser acupuncture) versus manual acupuncture for chronic insomnia: protocol for a randomized, assessor-blinded, superiority trial
Source: Front Psychiatry. 2026 Jun 1;17:1814245. doi: 10.3389/fpsyt.2026.1814245 (PMC13265499; doi:10.3389/fpsyt.2026.1814245)
Supplement: Supplementary file 2 [file Table2.docx]

**Sample Size Calculation for the Randomized Controlled Trial of LLLT vs. Manual Acupuncture**

**Calculation Method:**

Two-sample t-test with unequal variance

Power = 0.80, Alpha = 0.05

Based on pilot study post-treatment mean ISI = 10.9, acupuncture group = 13.4

SD = 4.44 (LLLT), 3.9 (Control), difference = 2.5

**Result:**

Required N = 90 (45 per group)

With 15% dropout rate → Total = 106 (53 per group)

PASS 2021, v21.0.3 2025/1/18 20:20:41 1

**Two-Sample T-Tests Allowing Unequal Variance**

**Numeric Results for an Unequal-Variance T-Test ───────────────────────────────────────**

δ = μ1 - μ2

Hypotheses: H0: δ = 0 vs. H1: δ ≠ 0

**Target Actual**

**Power Power N1 N2 N μ1 μ2 δ σ1 σ2 Alpha**

0.8 0.80129 45 45 90 10.9 13.4 -2.5 4.44 3.9 0.05

"Assuming a 15% dropout rate, a total of 106 participants will be required (90 / 0.85 = 106), with 53 participants in each group."
